# Numerical Optimization of Prednisolone–Tacrolimus Loaded Ultraflexible Transethosomes for Transdermal Delivery Enhancement; Box–Behnken Design, Evaluation, Optimization, and Pharmacokinetic Study

**DOI:** 10.3390/gels9050400

**Published:** 2023-05-10

**Authors:** Munerah M. Alfadhel, Randa Mohammed Zaki, Basmah Nasser Aldosari, Ossama M. Sayed

**Affiliations:** 1 Department of Pharmaceutics, College of Pharmacy, Prince Sattam Bin Abdulaziz University, P.O. Box 173, Al-Kharj 11942, Saudi Arabia; m.alfadhel@psau.edu.sa; 2 Department of Pharmaceutics and Industrial Pharmacy, Faculty of Pharmacy, Beni-Suef University, Beni-Suef 62514, Egypt; 3 Department of Pharmaceutics, College of Pharmacy, King Saud University, P.O. Box 2457, Riyadh 11451, Saudi Arabia; baldosari@ksu.edu.sa; 4 Department of Pharmaceutics, Faculty of Pharmacy, Sinai University-Kantara Branch, Ismailia 41612, Egypt; osama.sayed@su.edu.eg

**Keywords:** prednisolone, tacrolimus, Box–Behnken design, transdermal delivery, transethosomes

## Abstract

The aim of the present study is to formulate highly permeable carriers (i.e., transethosomes) for enhancing the delivery of prednisolone combined with tacrolimus for both topical and systemic pathological conditions. A Box–Behnken experimental design was implemented in this research. Three independent variables: surfactant concentration (X1), ethanol concentration (X2), and tacrolimus concentration (X3) were adopted in the design while three responses: entrapment efficiency (Y1), vesicle size (Y2), and zeta potential (Y3) were investigated. By applying design analysis, one optimum formulation was chosen to be incorporated into topical gel formulation. The optimized transethosomal gel formula was characterized in terms of pH, drug content, and spreadability. The gel formula was challenged in terms of its anti-inflammatory effect and pharmacokinetics against oral prednisolone suspension and topical prednisolone–tacrolimus gel. The optimized transethosomal gel achieved the highest rate of rat hind paw edema reduction (98.34%) and highest pharmacokinetics parameters (Cmax 133.266 ± 6.469 µg/mL; AUC_0-∞_ 538.922 ± 49.052 µg·h/mL), which indicated better performance of the formulated gel.

## 1. Introduction

For many years, prednisolone (PRED) has been utilized extensively in the treatment of inflammatory illnesses (both acute and chronic). For many rheumatic illnesses, a long-term remedy with these medications is frequently required to manage the symptoms. Long-term use of PRED in therapy has a number of negative consequences on the heart and metabolism of bones. One of the most common adverse effects of long-term PRED use is bone loss. The anti-inflammatory effect of PRED is underpinned by very intricate processes that obstruct the operation of numerous systems. In addition to the risks connected to the prolonged use of PRED at a super-physiological dose, there are issues connected to stopping the steroid medication. Reduced unfavorable side effects would be extremely beneficial for therapeutic usage of PRED, which is the greatest anti-inflammatory medicine currently on the market. For patients, worry regarding their safety has always been the main barrier to the use of oral PRED [1,2]. One of the means to reduce the side effects of PRED is to shift the route of application to the transdermal route.

Recently, the name “trans-ethosomes” was given to lipid-based nanovesicles that include an edge activator and ethanol. The benefits of both ethosomes and transfersomes are present in transethosomes (TETSMs). TETSMs provide a number of advantages over other drug delivery methods because of the inclusion of these components. Ethanol improves medication penetration through the minute holes created in the stratum corneum as a result of fluidization by enhancing the lipid’s fluidity and decreasing the density of the lipid bilayer [3]. These vesicles’ edge activator weakens the phospholipid bilayer, making them ultradeformable and extremely elastic [4]. Therefore, it is anticipated that these drug-loaded nanovesicles will have a positive effect on therapeutic activity.

Tacrolimus (TAC) alters the humoral and cell-mediated immune reactions linked to inflammation in a number of different ways. The key method of action involves reducing the immunophilin FK506 binding protein 12’s ability to activate calcineurin as a phosphatase, preventing the generation of interleukin (IL)-2, and inhibiting the signal transduction pathway that activates T cells. Although cyclosporin also inhibits calcineurin by forming a compound with a different immunophilin, in vitro and in vivo research shows that TAC has 10–100 times greater immunosuppressive activity than cyclosporin. Additionally, nitric oxide synthase activation and apoptosis may be inhibited by TAC, which may also enhance the effects of corticosteroids in these processes [5].

Many sources in the literature confirmed that combining corticosteroids therapy with TAC leads to lower required doses of corticosteroids in renal transplant operations [6]. This could be beneficial in terms of fewer side effects from steroids.

The aim of this research is to examine the possibility of combining PRED and TAC in TETSMs carriers and utilizing the transdermal route to increase the anti-inflammatory effect and pharmacokinetics of PRED.

## 2. Results and Discussion

### 2.1. Effect of Formulation Variables on EE%, Vesicle Size, and Zeta Potential

The chief objective of this study was to assess the effectiveness of TETSMs in improving the penetration of PRED through the skin for the management of inflammatory disorders. TETSMs are capable of effectively penetrating the stratum corneum due to their high alcohol content and surfactant properties [7]. Additionally, the study also examined the impact of TAC on the delivery of PRED to the skin.

An analysis was conducted to examine the impact of various formulation variables, including concentration of surfactant (X1), concentration of ethanol (X2), and concentration of TAC (X3) on EE% (Y1), vesicular size (Y2), and zeta potential (Y3). The results of the regression analysis, which were used to determine the best fitting model for each response, are summarized in Table 1.

#### 2.1.1. Effect of Formulation Variables on EE%

**Figure 1:**
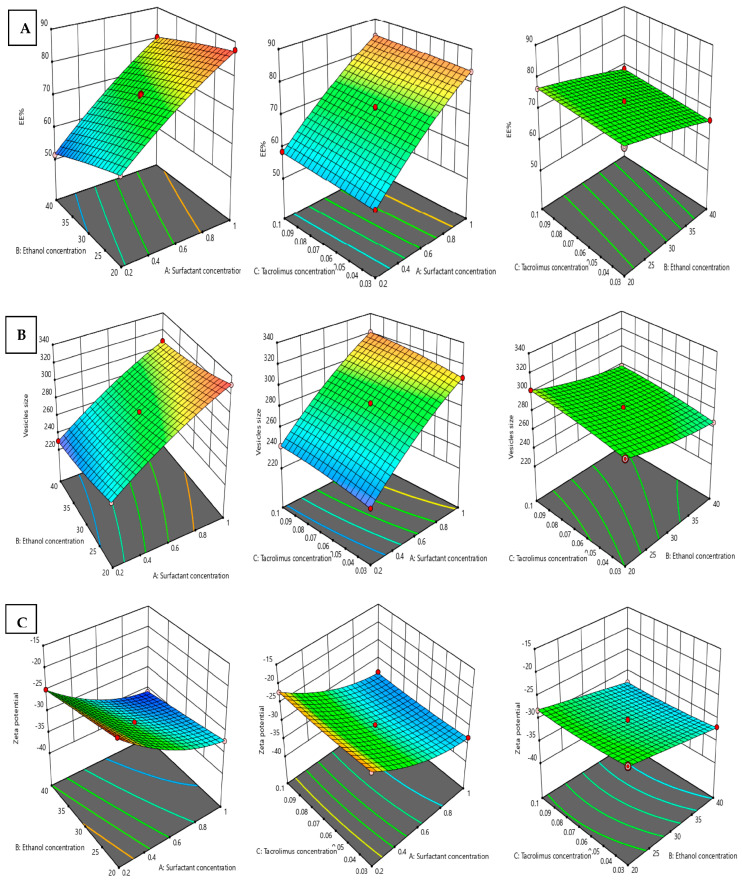
A 3D response surface graph for the effect of independent factors: surfactant concentration, Ethanol concentration, and Tacrolimus concentration on the dependent responses, EE% (**A**), vesicle size (**B**), and zeta potential (**C**) of PRED–TAC-loaded TETSMs. Red dots indicate the replicates in our design.

**Figure 2:**
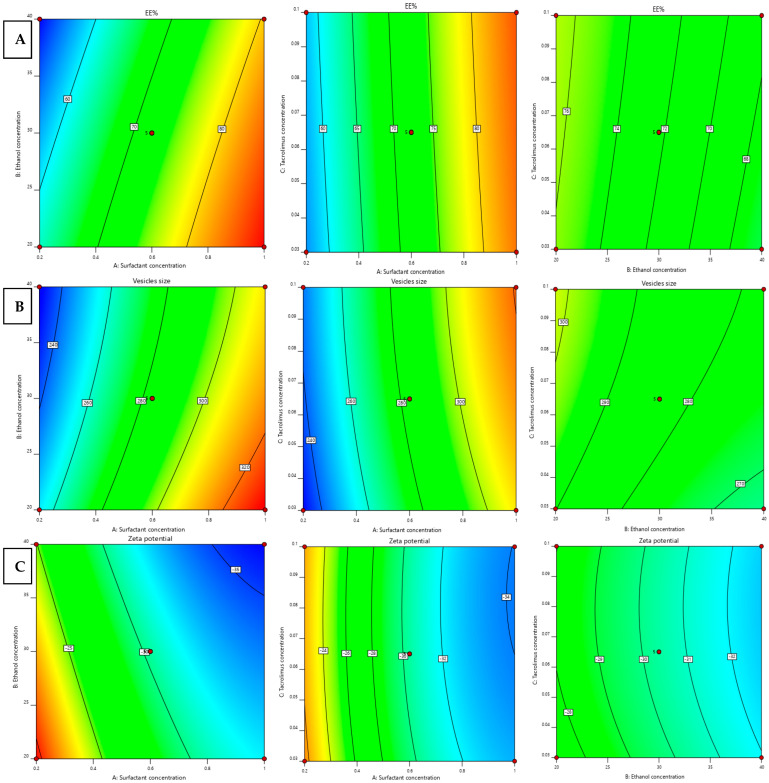
Contour graph for the effect of independent factors on different responses, EE% (**A**), vesicle size (**B**), and zeta potential (**C**) of PRED–TAC-loaded TETSMs. Red dots indicate the replicates in our design.

EE% data of the formulated TETSMs are shown in Table 1. Model fitting showed a fairly good fit with a quadratic interaction model, with a correlation coefficient R^2^ of 0.9994, adjusted R^2^ of 0.9986, and predicted R^2^ of 0.9932. The small difference (less than 0.2) between the adjusted and predicted R^2^ confirms the validity of the model [8]. Additionally, the model showed high adequate precision of 125.84 (greater than 4), indicating the ability of the model to navigate the design space [9], as represented in Table 2. ANOVA in Table 3 showed that EE% was significantly affected by all independent variables (*p* < 0.05) with the following equation representing the combined effect of independent variables on EE% of TETSMs:

(1) EE% =+72.26+13.35X1−4.40X2+0.7500 X3+0.9750X1X2−0.1250X1X3+0.3250X2X3−1.49X12−0.3425 X22−0.0925X32

where X1 is the concentration of surfactant, X2 is the concentration of ethanol, and X3 is the concentration of TAC. The response surface curves of the EE% values from the interaction of different independent variables are shown in Figure 1A and Figure 2A.

Increasing the surfactant concentration led to an increase in EE% of PRED as confirmed by the positive sign of the correlation coefficient in Equation (1). This could be explained based on the low HLB value of span 60, which increases the lipophilic domain of the lipid bilayer and hence increases the entrapped PRED in this hydrophobic domain [10,11].

Ethanol concentrations have a negative effect individually on the EE% of PRED in TETSMs, as represented by the negative sign of the correlation coefficient in Equation (1). The decrease in the EE% may be due to the increase in the fluidity and the leakage of the vesicles [12,13,14,15]. This result is in contrast to the previous research, which reported that enhancing the concentration of ethanol from 20 to 40% will have a positive impact on the EE% [16]. In addition, TAC showed an improvement in EE% by increasing its concentration from 0.03 to 0.1%, which aids the solubilization of drugs in the lipid mixture. Including lipid soluble TAC could increase the solubility of PRED.

#### 2.1.2. Effect of Formulation Variables on TETSMs Vesicle Size

The vesicle sizes of different formulated TETSMs are shown in Table 1. Model fitting revealed a quadratic interaction between independent variables, with R^2^ of 0.9999, adjusted R^2^ of 0.9998, and predicted R^2^ of 0.9990. There was a small difference (less than 0.2) between the adjusted and predicted R^2^. Additionally, the model showed high adequate precision of 325.6, as represented in Table 2. The data from ANOVA in Table 3 revealed that vesicle size of different formulations was significantly affected by all independent variables (*p* < 0.05) with the following equation representing the combined effect of independent variables on vesicle size of TETSMs:

(2) Vesicles size =+283.32+38.64 X1−11.76X2+6.00X3+0.1250 X1X2+0.6500 X1X3+0.0000X2X3−5.87X12+3.13X22−2.00X32

where X1 is the concentration of surfactant, X2 is the concentration of ethanol, and X3 is the concentration of TAC. Figure 1B and Figure 2B show the surface response curves of the combined effects of prepared independent variables on vesicle size. Generally, the surfactant concentration range in ethosomal formulations is 0.2–1% [17]. It was observed that increasing the surfactant (span 60) ratio resulted in a slight or moderate increase in vesicle size. The positive effect of surfactant on the vesicle size agrees with the earlier outcomes and can be due to the reduction in the hydrophilic portion of the surfactant in the presence of low HLB surfactant at high concentrations [10,11]. In addition, TAC had a positive impact on the vesicle size values, meaning that increasing TAC concentration from 0.03 to 0.1% resulted in a simultaneous increase in vesicle size. This condition was observed parallel to the increase in span 60. In contrast, the concentration of ethanol was found to have a negative impact on the vesicle size values and this is confirmed by the negative sign of the correlation coefficient (X2) in Equation (2). This observation agrees with the earlier literature [18].

Ethanol has been found to be a potent penetration enhancer. Ethanol concentration has been reported in the literature to be in the range of 10–50% [7,19]. Enhancing the concentration of ethanol resulted in a decrease in vesicle size, as stated in the previous works [7,15,20,21,22,23,24,25,26,27,28,29,30,31,32].

#### 2.1.3. Effect of Preparation Variables on Zeta Potential

Zeta potential is an indicator for the stability of nanosystems as it gives information about the magnitude of attraction and repulsion between nanoparticles. As zeta potential values increase, the repulsion between nanoparticles increases, and therefore there is improvement in the system stability. As shown in Table 1, the zeta potential values of all TETSMs formulations ranged from −19.3 ± 1.17 to −36.1 ± 0.87, indicating high stability for the fabricated formulations.

The model fitting of the combined effects of TETSMs variables on zeta potential proposed a quadratic model with R^2^ of 0.9988, adjusted R^2^ of 0.9974, and predicted R^2^ of 0.9877. There was a small difference (less than 0.2) between the adjusted and predicted R^2^. In addition, the model showed high adequate precision of 89.53 as represented in Table 2.

The data from ANOVA in Table 3 revealed that the zeta potential of different formulations was considerably affected by all independent variables (*p* < 0.05) with the following equation representing the effect of independent variables on zeta potential of TETSMs:

(3) $$\mathrm{Zeta}\;\mathrm{potential}=+30.38+5.90X1+2.31X2+0.3375X3-0.4000X1X2+0.1000X1+0.0250\;X2X3\;-2.28X1^{2}-0.0025X2^{2}-0.4025X32$$

where X1 is the concentration of surfactant, X2 is the concentration of ethanol, and X3 is the concentration of TAC. As clear from the above equation, all independent variables caused an increase in the zeta potential values. Increasing the zeta potential is so beneficial. The increase in zeta potential values with the increase in span 60 concentration may be attributed to the low HLB value (hydrophilic lipophilic balance) of span 60 resulting in high adsorption of OH ions from the hydration medium on the nanoparticles causing an increase in zeta potential values [15].

The positive impact of ethanol concentration on zeta potential values may be due to the negative charge imparted by ethanol on the particles’ surfaces. Ethanol’s effect on zeta potential is well documented in the literature [17,33,34,35].

The effect of different independent variables on zeta potential is shown in Figure 1C and Figure 2C.

### 2.2. Formulation Optimization and Validation

**Figure 3:**
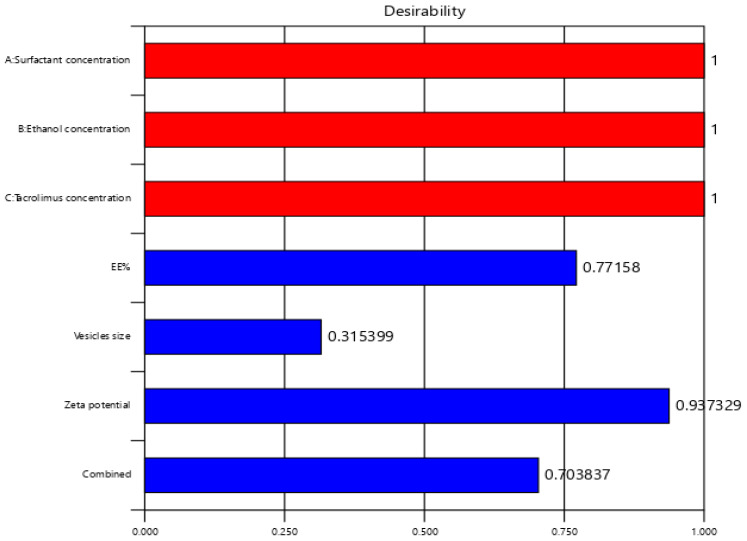

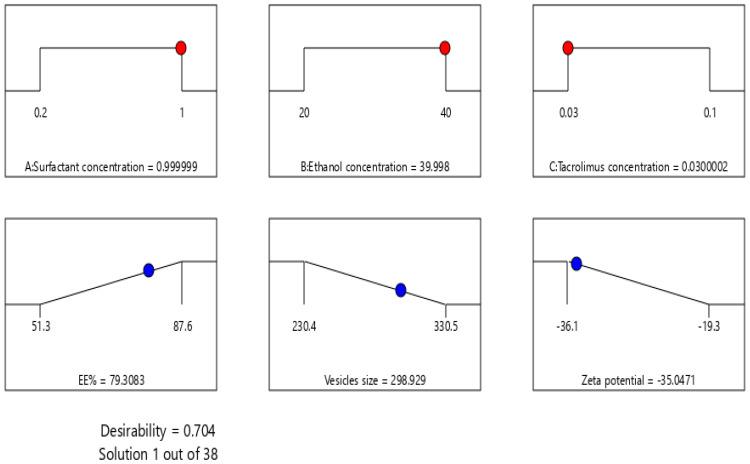
Desirability plot of the numerical optimization of PRED–TAC-loaded TETSMs.

A numeric optimization was performed by Design Expert software to select the optimum formula based on the highest desirability value. The optimum formula was found to consist of 0.999 (*w*/*v* %) span 60, 39.99 (*v*/*v* %) ethanol, and 0.03 (*w/v* %) TAC. The predicted responses of EE%, vesicle size and zeta potential were 79.308%, 298.929 nm, and −35.047 mV, respectively, with a desirability of 0.704 as represented in Table 4 and Figure 3. Validation of the optimum formula resulted in a % relative error less than 5% for all predicted responses, confirming the fitness of the model [36].

### 2.3. In Vitro Release of Prednisolone from Optimized TETSMs Containing Gel

**Figure 4:**
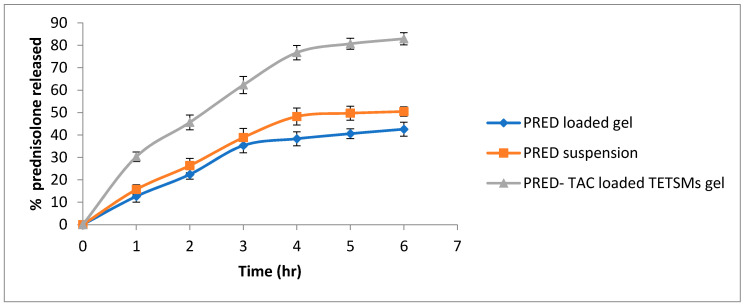
In vitro release profile of PRED from the optimum PRED–TAC-loaded TETSMs gel compared to PRED suspension and the PRED-loaded gel.

The release profile of PRED is illustrated in Figure 4. The release profile showed that the PRED–TAC-loaded TETSMs gel achieved higher released PRED (82.93% ± 2.75) than the PRED-loaded gel (42.56% ± 3.11) and PRED suspension (50.45% ± 2.12). The higher release achieved could be attributed to the increased thermodynamic activity of PRED solubilized in the TETSMs lipid bilayers, and to the nanosize of the vesicles that led to higher release [37]. All gel formulations exhibited Higuchi diffusion model release.

### 2.4. PRED Permeation from Optimized Formula

**Figure 5:**
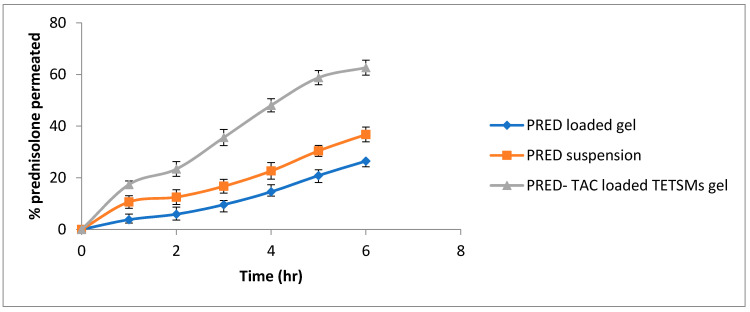
Ex vivo permeation of prednisolone from the optimum-formula-loaded gel compared to PRED suspension and the PRED-loaded gel.

The permeation profile of PRED from the optimized-formula-loaded gel compared to PRED-loaded gel and PRED suspension is illustrated in Figure 5, while permeation parameters are presented in Table 5. Preparations of PRED–TAC-loaded TETSMs gels showed the highest percent of PRED (62.65% ± 2.87) permeated through the skin, compared to PRED-loaded gels and PRED suspension (26.5% ± 2.21 and 36.76% ± 1.98, respectively), and this is due to the lower rigidity of the vesicles, which permits their squashing between cells. The combined effect of the presence of both ethanol and edge activator (i.e., surfactant) enhanced the transepidermal flux of the gel formulation (45.27± 2.87 × 10^4^ µg/cm^2^·h^−1^) compared to the PRED-loaded gels and PRED suspension.

### 2.5. Morphological Examination of PRED–TAC-Loaded TETSMs

**Figure 6:**
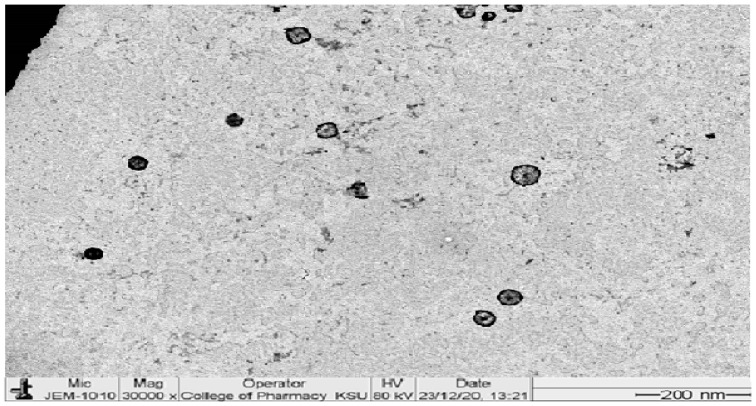
TEM image of the optimum formula.

The transmission electron image of the optimum formula is represented in Figure 6. The image shows spherical vesicles with large aqueous cores. The vesicles are smooth, well defined, and non-aggregated, confirming their stability. The image is also in agreement with the size analysis data.

### 2.6. Characterization of Gel Formulations

Gel formulations of PRED were characterized regarding pH, % drug content, and spreadability. The data of these characterizations are shown in Table 6. Regarding pH, all formulations showed a pH around 6.5 which is dermatologically acceptable according to the literature [38]. All formulations kept a good % drug content meaning good compatibility of the mixed ingredients.

The spreadability has a substantial role in patient agreement and aids in uniform gel administration to the skin. An acceptable gel needs less time to spread over the skin and will have great spreadability. The values of spreadability (4.23–3.87 (g cm)/s) confirm that the gels spread without difficulty by application of a sheer minimum and have satisfactory bioadhesion [39].

### 2.7. In Vivo Evaluation of Anti-Inflammatory Effect of PRED Containing TETSMs Gels

**Figure 7:**
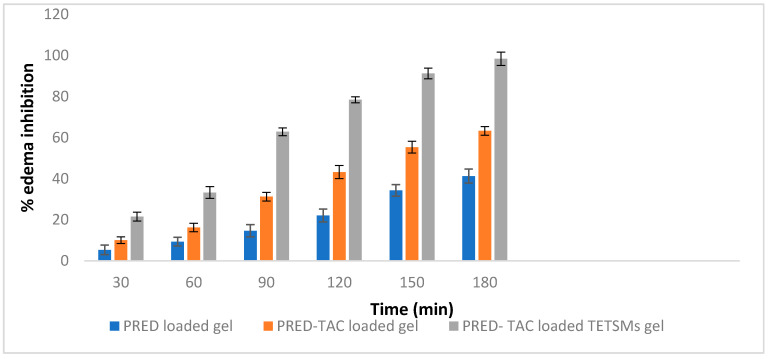
% Edema inhibition of PRED gel formulations.

The rat hind paw edema model is one of the most used anti-inflammatory models to study the effect of topically applied anti-inflammatory agents. The percent edema inhibition of PRED-loaded gel, PRED–TAC-loaded gel, and PRED–TAC-loaded TETSMs gel are shown in Table 7 and Figure 7. It was noted that PRED–TAC-loaded TETSMs gel achieved the highest rate of percent inhibition (*p* < 0.05) among the other gel formulations (98.34% in 180 min), followed by PRED–TAC-loaded gel (63.26%), and PRED-loaded gel (41.25%).

PRED, as one of the corticosteroids, has the ability to suppress every step of the inflammatory cascade including synthesis of inflammatory mediators and cell-mediated immunity [40]. The TETSMs gel formulation enhanced the permeation of PRED and its localization in skin layers due to both ethanol and edge activator (surfactant) effects. Additionally, TAC has a synergistic effect on the inflammatory process due to its effect on inhibiting the production of cytokines involved in the inflammatory cascade [41,42].

### 2.8. Comparative Pharmacokinetic Study of Prednisolone Gel Formulations against Oral PRED Suspension

**Figure 8:**
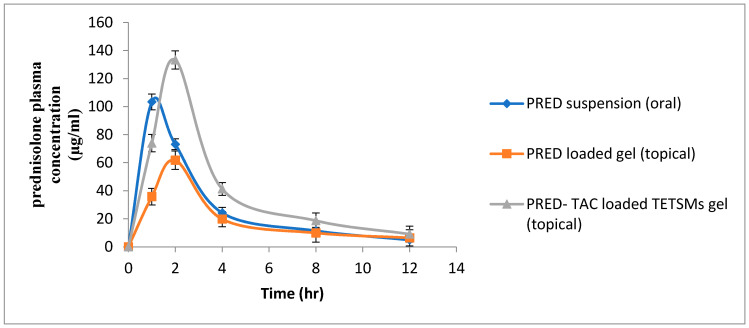
Plasma concentration time profiles of PRED formulations.

Pharmacokinetic parameters of PRED-containing gel formulations and oral PRED suspension are shown in Table 8. Plasma PRED profiles are illustrated in Figure 8. It is noticed that PRED–TAC-loaded TETSMs gel achieved higher pharmacokinetic parameters (*p* < 0.05) compared to PRED-loaded gel (topical) and PRED oral suspension in terms of Cmax and AUC 0-∞ (133.266 ± 6.469 µg/mL, 538.922 ± 49.052 µg·h/mL), (1.3 and 2.2-fold increase in Cmax and 1.47 and 1.88 fold increase in AUC 0-∞ compared to oral suspension and PRED gel, respectively), with significance (*p* < 0.05) according to ANOVA results in Table 9. The higher systemic concentration of PRED after application of PRED–TAC-loaded TETSMs gel than after oral administration could be attributed to the presence of edge activators in the TETSMs composition which increase skin penetration by enhancing the fluidity of the TETSMs phospholipid bilayer, making them ultradeformable and extremely elastic, and therefore facilitating their squeezing into the skin pores [43,44]. Additionally, the presence of ethanol in TETSMs improves drug penetration through the minute holes created in the stratum corneum as a result of fluidization by enhancing the lipids fluidity and decreasing the density of the lipid bilayer [11]. Moreover, the addition of TAC to TETSMs gel formulations resulted in higher pharmacokinetic parameters of PRED, as TAC is previously reported to increase corticosteroids accumulation [45]. These findings support the fact that transdermal delivery bypasses first-pass metabolism for many active ingredients including corticosteroids.

## 3. Conclusions

The present research proved that incorporating TAC with PRED in TETSMs and transdermal application increased the efficacy and optimized the pharmacokinetics of PRED, thus improving the efficacy of the therapy for both topical and systemic pathological conditions. These findings focus the spotlight on a way to decrease the proposed doses of corticosteroids, utilizing the benefits of both changing the route of administration and the incorporation of TAC. This will lead to better management of inflammatory conditions and fewer side effects linked to the use of corticosteroids.

## 4. Material and Methods

### 4.1. Materials

Prednisolone (PRED) and tacrolimus (TAC) were kindly gifted by Aljazeera Pharmaceuticals Company. Phosphatidylcholine from soy lecithin (Phospholipon 90G, pure phosphatidylcholine stabilized with 0.1% ascorbyl palmitate and max 0.3% tocopherol) was a gift from LIPOID GmbH, Germany. Ethanol, span 60, and hydroxypropyl methyl cellulose were purchased from Sigma-Aldrich.

### 4.2. Experimental Design Setup

A Box–Behnken design was utilized to optimize the formulation variables for creating PRED-loaded TETSMs with the goal of achieving high encapsulation efficiency, small vesicle size, and high zeta potential. Experiments were conducted using Design Expert software, with 17 total experiments conducted, including 13 at the midpoint of each edge of a multidimensional cube and 4 replicates of the cube’s center point. Three independent variables were estimated, including surfactant concentration, ethanol concentration, and tacrolimus concentration. The dependent variables were encapsulation efficiency, vesicle size, and zeta potential. The study design and composition of the prepared PRED-loaded TETSMs are presented in Table 1 and Table 10. The optimized formula was selected by Design Expert software based on the highest value of desirability for achieving our goal of high encapsulation efficiency, high zeta potential, and small particle size. The selected optimized formula was fabricated and assessed again for different responses to detect the accuracy of the model.

### 4.3. Preparation of PRED-Loaded TETSMs

Fabrication of PRED-loaded TETSMs was performed according to the thin film hydration method [46]. Briefly, the accurate weight of phosphatidylcholine, PRED (100 mg), TAC, and span 60 were dissolved in 10 mL organic solvent, which consisted of 2:1 chloroform: methanol in a rounded flask. Then the organic solvent was evaporated under vacuum using a rotary evaporator (Buchi Rotavapor R-200, Switzerland) at 60 °C and 100 rpm. The formed film was then hydrated with 10 mL of a certain ethanol concentration as specified in Table 2. The hydration step was carried out at 60 °C to be higher than the lipid transition temperature [11]. Finally, the formulated dispersions were kept in a refrigerator for further analysis.

### 4.4. Characterization of Formulated PRED-Loaded TETSMs

#### 4.4.1. Entrapment Efficiency Estimation

The entrapment efficiency (EE%) of PRED in the fabricated preparations was calculated by determining the percentage of PRED found in the fabricated preparations. To separate unentrapped drug, cooling centrifugation was performed at 16,000 rpm and 4 °C using a Sigma cooling centrifuge from Sigma Laborzentrifugen GmbH. The clear supernatant was then analyzed for PRED content at λ max 254 nm using a Jasco UV–Vis spectrophotometer from Jasco, Japan. The EE% can be computed by subtraction of the quantity of PRED present in the supernatant from the initial quantity of PRED added [47].

#### 4.4.2. Vesicle Size and Zeta Potential Analysis

The zeta potential, polydispersity index (PDI), and average vesicle size of all fabricated preparations were measured using photon correlation spectroscopy with a Zetasizer 2000 (Malvern Instruments Ltd., Malvern, UK) [48]. The nanodispersions were exposed to dilution before analysis and experiments were conducted in triplicate at a 90-degree scattering angle and a temperature of 25 degrees Celsius prior to taking measurements.

#### 4.4.3. Formulation Optimization

The optimized formulation for PRED was selected using Design Expert^®^ software (Ver. 12, Stat-Ease, Minneapolis, MN, USA), with the goal of achieving the highest EE% and zeta potential, while minimizing vesicle size. The selected optimized formulation was then fabricated and tested in triplicate to validate the accuracy of the model by comparing the predicted responses from the software with actual measurements, using the equation for relative error [49,50].

% Relative error = ((predicted value − actual value)/predicted value) × 100(4)

### 4.5. Preparation of Gels Containing Optimized TETSMs:

The optimized formula was amalgamated into a gel base using hydroxypropyl methylcellulose (HPMC, K4M) as a gelling agent at 2.5% (*w*/*v*). Briefly, the accurate weight of HPMC (0.25 g) was dispersed in 10 mL distilled water while stirring at 1000 rpm to obtain homogenous dispersion. Then the optimum TETSMs formula was dispersed into the gel base with continuous stirring to obtain a final gel formulation with 5 mg PRED per 1 g gel [36].

### 4.6. Evaluation of Gel Formulations

#### 4.6.1. pH Evaluation

A 100 mg sample of the TETSMs-loaded gel formulation was weighed and added to a 50 mL volumetric flask. The volume was then filled to 50 mL with double-distilled water. The pH of the HPMC gel loaded with TETSMs was noted down using a glass microelectrode (Mettler Instruments, Giessen, Germany) by measuring the pH after 1 min of equilibration. The experiments were repeated three times to confirm the neutralization of the gel from different batches. The pH was measured on the first, 15th, and 30th day after preparation to check for any changes in pH over time.

#### 4.6.2. Spreadability

A modified instrument called the spreadability apparatus was used to test spreadability. It consisted of two glass slides with gel in the middle, with the upper slide attached to a balance by a hook and the lower slide fixed to a wooden plate. On the basis of the gel’s slip and drag characteristics, spreadability was measured [51]. Spreadability was computed using the following equation:

s = m × l/t(5)

where s represents the spreadability, m is the weight in pan (g), l is the fixed distance moved by the slide and t is the time.

#### 4.6.3. Drug Content Determination

The created gels were given 48 h to rest before 1 g of each was taken in a 10 mL volumetric flask, dissolved in methanol, and the remaining 10 mL was filled with water. For PRED, maximum absorbance values were determined spectrophotometrically using the Jasco V530, Tokyo, Japan at 254 nm. From a standard calibration curve in methanol, concentrations of PRED were determined [52].

#### 4.6.4. In Vitro Drug Release Studies

The release of PRED from the PRED–TAC-loaded TETSMs gel, PRED-loaded gel, and PRED suspension was tested in vitro using a dialysis bag method. An amount of 3 mg of PRED was placed in a dialysis bag with a molecular weight cut-off of 12,000 Da and released into 50 mL of Sorensen’s phosphate buffer at pH 7.4. Samples were taken from the medium at various intervals and replaced with fresh medium over a period of 6 h [53]. The samples were analyzed using spectrophotometry at a wavelength of 254 nm (using a Jasco V530 from Tokyo, Japan). The release kinetics were determined by applying Korsmeyer–Peppas, Zero-order, First-order, Higuchi, and Hixson models to the data obtained.

#### 4.6.5. In Vitro Permeation Studies

The barrier membrane samples of hairless rat skin were used to conduct the study after approval by the Institutional Animal Ethical Committee (IAEC) number SCBR-055-2022, Prince Sattam Bin Abdulaziz University. Firstly, the animals were anesthetized by ether and sacrificed; then the abdominal hair was cautiously shaved off. Secondly, skin samples were excised, and the subcutaneous tissues were detached carefully and finally washed with saline and stored at −20 °C until use. The membrane samples were placed in vertical Franz diffusion cells, consisting of a receptor compartment with a capacity of 5 mL and a diffusion surface area of 1 cm^2^. Isotonic phosphate buffer saline (1.55 M, pH 7.4) was placed in the receptor compartments and stirred at 600 rpm in a temperature-controlled water bath. Before starting the experiment, phosphate buffer saline was added to the donor compartment at 37 °C to equilibrate the biological membranes for thirty minutes. After this time, the phosphate buffer saline was cautiously detached from the donor compartment. Afterward, a suitable quantity of TETSMs gel equivalent to 3 mg PRED was added to the donor compartment and spread over the membrane. To maintain occlusive conditions in the skin, glass slips were put on the donor compartments during the experiment. The in vitro permeation studies were conducted for 6 h and, at different time intervals, 1 mL samples were withdrawn from the receptor compartment and substituted with the same quantity of buffer. All experiments were repeated five times [53,54].

The cumulative amount permeated was computed using Equation (3) by taking into account the volume of the receptor phase (V_R_), the volume of the sample at each time point (V_s_), and the quantified concentration of the sample taken at the nth time point (C_n_). This cumulative amount calculated at each time point was then divided by the diffusion area of the Franz cells (measured in cm^2^) to obtain the final result.

(6) $$\mathrm{Cumulative}\;\mathrm{amount}=V_{R}\times {\;C}_{n}+\lfloor V_{s}\left(\sum C_{1}+\cdots +C_{n-1})\right\rfloor$$

#### 4.6.6. Data Analysis for Permeation Studies

The different parameters of the skin permeation test were studied and assessed. The steady state flux (*J_ss_*) of the drug in μg·cm^−2^·h^−1^ at time t was computed from the slope of the linear portion of the plot representing the cumulative amount of PRED permeated per unit area over time. The cumulative amount of PRED in the receptor compartment after 6 h was known as Qcum (μg·cm^−2^). The permeability coefficient (*k_p_*) of PRED in each formulation was computed by dividing the steady state flux (*J_ss_)* by the initial concentration of PRED in the donor compartment (which is considered the saturated solubility of PRED in each formulation) (*C_o_*) as presented in Equation (4). The results were represented as mean ± S.D. [55].

(7) $$\;\;\;k_{p}=\frac{J_{ss}}{C_{o}}$$

ANOVA was used to analyze the data of flux and to compare the data of flux with the cumulative amount of the drug in the receptor compartment of different formulations, followed by a Tukey’s honestly significant difference test using IBM SPSS^®^ version 23 for Windows^®^. A probability of less than 5% (*p* < 0.05) was considered significant.

### 4.7. In Vivo Estimation of Anti-Inflammatory Effect of PRED-Loaded TETSMs Gels

Using adult male albino rats, the anti-inflammatory effectiveness of the best formulation of PRED–TAC-loaded TETSMs gel was compared to PRED–TAC-loaded gel and PRED-loaded gel. The study was conducted according to the guidelines of the Declaration of Helsinki and approved by the Institutional Animal Ethical Committee (IAEC) number SCBR-055-2022 of CPCSEA (Committee for Control and Supervision of Experiments on Animals), Prince Sattam Bin Abdulaziz University. Rats ranged in weight from 100 to 185 g. Before beginning the tests, they were housed for a week at the animal house at 25 ± 2 °C and a light: dark cycle of 12:12 h. Rats were fed a conventional rat pellet diet, which was removed 12 h before the experiment, although water was still available. All studies were conducted in accordance with the ethical standards for research using experimental animals as well as the guidelines for the care of laboratory animals. In the present investigation, 24 rats were separated into four groups of six rats each (n = 6), and the following study protocols were used.

First group: served as a positive control and received PRED-loaded gel.

Second group: received PRED–TAC-loaded gel.

Third group: received PRED–TAC-loaded TETSMs gel.

Fourth group: served as a negative control group.

#### 4.7.1. Carrageenan-Induced Acute Inflammation

By using carrageenan-induced paw edema, the anti-inflammatory effect of the best formulation of PRED–TAC-loaded TETSMs gel was compared to PRED–TAC-loaded gel and PRED-loaded gel. The right paw of each animal was massaged 50 times with 0.5 g of each treatment’s optimal formulation of PRED–TAC-loaded TETSMs gel, PRED–TAC-loaded gel, and PRED-loaded gel an hour before carrageenan administration to allow formulation penetration through the skin. Acute inflammation was generated in all groups an hour after treatment by injecting 0.1 mL of 1% *w*/*v* carrageenan saline solution into each rat’s right hind paw’s sub-plantar tissue. Following carrageenan administration, the rats were monitored for three hours [56,57].

#### 4.7.2. Comparative Pharmacokinetics

Blood samples of 0.5 mL were collected at 1, 2, 4, 8, and 12 h from the abdominal aorta after application of oral PRED suspension, topical PRED-loaded gel, and topical PRED–TAC-loaded TETSMs gel at a dose of 50 mg/kg. To separate the plasma from different blood samples, all blood samples were centrifuged at 2000× *g* for 15 min. Then plasma was instantaneously conveyed into clean tubes and stored at –20 °C for further analysis.

### 4.8. HPLC Conditions

#### HPLC assay of Prednisolone in Plasma

To prepare the plasma samples for the HPLC assay, all samples were vortexed; then aliquots of 300 μL of plasma were put into falcon tubes. After that, 40 μL of 1 μg/mL dexamethasone (internal standard) was added to the plasma samples. For Prednisolone extraction, 1 mL of ethyl acetate was added and vortexed for 10 min then centrifuged at 2500× *g* for 10 min (4 °C) to allow phase separation. Finally, the upper layer was transferred to a glass tube and evaporated at 45 °C to dryness then reconstituted with 300 μL of mobile phase and injected into the HPLC system.

For quantitative determination of prednisolone in plasma samples, 100 µL aliquots were injected in a Shimadzu HPLC system (SHIMADZU 1200 series HPLC system (Kyoto, Japan) equipped with a quaternary pump, an online degasser, and an autosampler (SHIMADZU1200, Kyoto, Japan). a Ther-mosil^®^ C-18 column (250 mm × 4.6 mm i.d., 5 µm particle size) was used and operated at 30 °C. The system was equipped with UV–Vis detectors set at 254 nm. Isocratic elution was performed using acetonitrile and water (36:64) as the mobile phase (1) with a flow rate of 1.2 mL/min and a total time of 12 min. The liquid chromatography instrument was interfaced with a computer software using Microsoft Windows 7 [58].

### 4.9. Pharmacokinetic Analysis

Pharmacokinetic parameters were calculated using WinNonlin software (version 1.5, Scientific Consulting, Inc., Rockville, MD, USA). Pharmacokinetic parameters include C max (maximum plasma concentration), Tmax (time required for peak concentration), AUC (area under the curve), T_1\2_ (half-life), and MRT (mean residence time). The obtained parameters were subjected to analysis by ANOVA and Fisher’s PSLD test for multiple evaluations among groups. Results were considered significant if the *p* value was less than 0.05. All results were reported as the mean ± SD.

## Figures and Tables

**Table 1:** Composition of PRED-TAC TETSMs Formulations and the results of different responses (mean ± SD).

Formula Code	Independent Variables			Dependent Variables			
	Surfactant Concentration *w*/*v* % (X1)	Ethanol Concentration *v*/*v* % (X2)	Tacrolimus Concentration *w*/*v* % (X3)	EE% (Y1)	Vesicles Size (nm) (Y2)	Zeta Potential (mv) (Y3)	PDI
1	1	20	0.065	87.6 ± 2.14	330.5 ± 10.12	−32.2 ± 0.92	0.132 ± 0.08
2	1	40	0.065	80.4 ± 1.87	307.8 ± 8.75	−36.1 ± 0.87	0.315 ± 0.09
3	1	30	0.1	84.4 ± 1.46	320.7 ± 7.35	−33.8 ± 1.01	0.326 ± 0.11
4	0.2	20	0.065	62.4 ± 2.01	253.6 ± 6.54	−19.3 ± 1.17	0.321 ± 0.12
5	0.6	30	0.065	72.3 ± 3.27	283.5 ± 5.43	−30.4 ± 1.28	0.276 ± 0.03
6	0.6	30	0.065	72.1 ± 2.98	283.2 ± 6.98	−30.1 ± 0.84	0.376 ± 0.11
7	0.2	30	0.1	58.4 ± 1.54	242.1 ± 4.33	−22.1 ± 0.58	0.123 ± 0.02
8	0.6	30	0.065	72.4 ± 1.63	282.9 ± 7.21	−30.3 ± 0.23	0.265 ± 0.06
9	0.2	30	0.03	56.7 ± 3.21	231.5 ± 3.75	−21.8 ± 0.67	0.225 ± 0.14
10	0.6	20	0.03	75.6 ± 2.19	290.4 ± 7.25	−27.3 ± 0.95	0.431 ± 0.10
11	0.6	30	0.065	72.6 ± 1.92	283.7 ± 3.98	−30.5 ± 0.26	0.259 ± 0.07
12	0.6	20	0.1	76.5 ± 1.64	302.6 ± 9.43	−28.1 ± 1.11	0.239 ± 0.13
13	0.6	40	0.1	68.7 ± 2.54	278.5 ± 5.38	−32.7 ± 0.83	0.185 ± 0.05
14	0.2	40	0.065	51.3 ± 2.73	230.4 ± 6.27	−24.8 ± 0.63	0.173 ± 0.16
15	0.6	30	0.065	71.9 ± 3.81	283.3 ± 4.79	−30.6 ± 0.27	0.265 ± 0.08
16	1	30	0.03	83.2 ± 1.26	307.6 ± 10.3	−33.1 ± 1.03	0.439 ± 0.14
17	0.6	40	0.03	66.5 ± 1.87	266.7 ± 7.83	−31.8 ± 0.68	0.286 ± 0.12

**Table 2:** Output data of Box–Behnken design for optimization of PRED–TAC-loaded TETSMs.

Dependent Variables	R^2^	Adjusted R^2^	Predicted R^2^	Adequate Precision
Y1: % EE	0.9994	0.9986	0.9932	125.8424
Y2: Vesicle size (nm)	0.9999	0.9998	0.9990	325.6015
Y3: Zeta potential (mV)	0.9988	0.9974	0.9877	89.5319

**Table 3:** ANOVA for Box–Behnken design of PRED–TAC-loaded TETSMs.

Dependent Variable	Source	SS	Df	Mean Square	F Value	*p* Value	
Y1	Model	1599.74	9	177.75	1313.87	<0.0001	significant
	A-Surfactant concentration	1425.78	1	1425.78	10,539.03	<0.0001	
	B-Ethanol concentration	154.88	1	154.88	1144.84	<0.0001	
	C-Tacrolimus concentration	4.50	1	4.50	33.26	0.0007	
	AB	3.80	1	3.80	28.11	0.0011	
	AC	0.0625	1	0.0625	0.4620	0.5185	
	BC	0.4225	1	0.4225	3.12	0.1205	
	A^2^	9.38	1	9.38	69.33	<0.0001	
	B^2^	0.4939	1	0.4939	3.65	0.0977	
	C^2^	0.0360	1	0.0360	0.2663	0.6217	
Y2	Model	13,537.78	9	1504.20	9232.25	<0.0001	significant
	A-Surfactant concentration	11,942.85	1	11,942.85	73,301.15	<0.0001	
	B-Ethanol concentration	1106.85	1	1106.85	6793.48	<0.0001	
	C-Tacrolimus concentration	288.00	1	288.00	1767.65	<0.0001	
	AB	0.0625	1	0.0625	0.3836	0.5553	
	AC	1.69	1	1.69	10.37	0.0146	
	BC	0.0000	1	0.0000	0.0000	1.0000	
	A^2^	145.21	1	145.21	891.22	<0.0001	
	B^2^	41.18	1	41.18	252.77	<0.0001	
	C^2^	16.80	1	16.80	103.11	<0.0001	
Y3	Model	345.94	9	38.44	671.83	<0.0001	significant
	A-Surfactant concentration	278.48	1	278.48	4867.32	<0.0001	
	B-Ethanol concentration	42.78	1	42.78	747.74	<0.0001	
	C-Tacrolimus concentration	0.9112	1	0.9112	15.93	0.0053	
	AB	0.6400	1	0.6400	11.19	0.0123	
	AC	0.0400	1	0.0400	0.6991	0.4307	
	BC	0.0025	1	0.0025	0.0437	0.8404	
	A^2^	21.84	1	21.84	381.72	<0.0001	
	B^2^	0.0000	1	0.0000	0.0005	0.9835	
	C^2^	0.6821	1	0.6821	11.92	0.0106	

Y1: % EE, Y2: Vesicle size (nm), Y3: Zeta potential (mV), SS: sum of squares, Df: degree of freedom.

**Table 4:** The composition and validation of the optimized formula with its expected responses.

The Optimized Formula	Independent Variables			Predicted Responses			Desirability
	Surfactant Concentration *w/v* % (X1)	Ethanol Concentration *v/v* % (X2)	Tacrolimus Concentration *w/v* % (X3)	EE%	Vesicle Size (nm)	Zeta Potential (mv)	0.704
	0.9999	39.998	0.03000	79.3083	298.929	−35.0471	
Validation of the Optimum Formula							
Responses	Predicted value		Experimental value		% Relative error		
EE%	79.3083		81.892		3.258		
Vesicle size (nm)	298.929		305.325		2.139		
Zeta potential (mv)	−35.0471		−34.46		1.675		

**Table 5:** Ex vivo permeation parameters of PRED from the optimum-formula-loaded gel compared to PRED suspension and PRED-loaded gel (n = 3, mean ± SD).

Formulation Code	Flux (J_ss_) (µg/cm^2^ h^−1^) × 10^4^	Permeability Coefficient (P) (cm/h) × 10^−6^	Partition Coefficient (K_P_) × 10^4^
PRED–TAC-loaded TETSMs gel	45.27 ± 2.87	9.21 ± 2.87	242 ± 2.87
PRED suspension	27.87 ± 2.43	5.11 ± 2.43	95.41 ± 2.43
PRED-loaded gel	19.32 ± 3.12	3.42 ± 3.12	38.54 ± 3.12

**Table 6:** Characterization of PRED containing gel formulations.

Formula	pH	%Drug Content	Spreadability
PRED–TAC-loaded TETSMs gel	6.92 ± 0.014	96.53 ± 1.34	4.23 ± 0.12
PRED-loaded gel	6.54 ± 0.015	98.23 ± 0.42	3.87 ± 0.23
PRED–TAC-loaded gel	6.73 ± 0.009	97.48 ± 1.51	3.92 ± 0.15

**Table 7:** % Edema inhibition of PRED gel formulations.

Time (min)	% Edema Inhibition		
	PRED-Loaded Gel	PRED–TAC-Loaded Gel	PRED–TAC-Loaded TETSMs Gel
30	5.34	10.12	21.56
60	9.34	16.24	33.25
90	14.67	31.27	62.87
120	22.11	43.24	78.43
150	34.32	55.32	91.2
180	41.25	63.26	98.34

**Table 8:** Pharmacokinetic parameters of PRED formulations (mean ± SD).

Pharmacokinetic Parameters	PRED Suspension (Oral)	PRED-Loaded Gel (Topical)	PRED–TAC-Loaded TETSMs Gel
Cmax	103.333 ± 5.686	61.7 ± 6.564	133.266 ± 6.469
Tmax	1.00 ± 0.000	2.00 ± 0.000	1.96 ± 0.057
t1/2	3.449 ± 0.413	4.926 ± 0.344	3.665 ± 0.428
AUC 0-t	341.080 ± 33.666	240.056 ± 36.342	490.233 ± 32.855
AUC 0-inf_obs	365.769 ± 42.054	285.776 ± 45.123	538.922 ± 49.052
MRT 0-inf_obs	4.120 ± 0.480	6.293 ± 0.348	4.852 ± 0.466

**Table 9:** One way ANOVA of pharmacokinetic parameters of PRED formulations.

Pharmacokinetic Parameters	SS	Df	MS	F	*p*-Value	F Crit
Cmax	7751.127	2	3875.563	99.13899	0.017047	5.143253
Tmax	1.935556	2	0.967778	871	0.001355	5.143253
t1/2	3.816098	2	1.908049	12.09148	0.007855	5.143253
AUC 0-t	95040.8	2	47520.4	40.34386	0.000332	5.143253
AUC 0-inf_obs	100463.8	2	50231.89	24.26347	0.001332	5.143253
MRT 0-inf_obs	7.33255	2	3.666275	19.28791	0.002439	5.143253

**Table 10:** Dependent and independent variables in Box–Behnken design.

Independent Variables	Levels	
	Low	High
Surfactant concentration *w*/*v* % (X1)	0.2	1
Ethanol concentration *v*/*v* % (X2)	20	40
Tacrolimus (TAC) concentration (X3)	0.03	0.1
Dependent values (Responses)	Desirability	
EE% (Y1)	maximize	
Vesicle size (Y2)	minimize	
Zeta potential (Y3)	maximize	

## Data Availability

The data is contained in the manuscript.
